# Air-Raids on Hospitals and Non-Combatants

**Published:** 1917-09-15

**Authors:** 


### AIR-RAIDS ON HOSPITALS AND NON-COMBATANTS.

The war policy of the German military organisation has long since revealed its motives and character, and has gradually succeeded in forcing the greater part of the civilised world to the conviction that, if life is to be worth living, this scheme of horror and cruelty must be destroyed. Protests and condemnations are, in the circumstances, but idle words. The remedy lies elsewhere, and, as may be judged from President Wilson's reply to the Pope's letter, nowhere is this necessity more clearly seen than in the United States. If any degree of hesitation on such a point still lingered it is likely to be removed by the latest expression of German barbarity as seen in the deliberate air-raids practised on hospitals containing wounded soldiers. The evidence is clear that these have been no accidental happenings. On the contrary, some of the hospitals attacked have been far behind the fighting line, and the intent has been evident from the manner of procedure.

It is significant that this wicked and cowardly policy has been received with comparative calm, so far at least as outward expression is concerned. So completely is it in harmony with what has gone before that it hardly elicits a word of surprise. The relative silence is a measure of the level to which the German character has fallen in the public judgment. The act is what well might be expected from such a source, and no condemnation could be more severe than the one implied in such a conviction. But though men are silent, these things most surely will not be forgotten, nor must they be forgotten until due punishment is enacted. Still more, the nation which approves these crimes against humanity can receive no welcome or even toleration from civilisation so long as she is unrepentant and unashamed. There is an appropriate word spoken by Burke: "If we do not hate what we ought to hate we cannot love what we ought to love."

Memories of similar character rest with us from events which have recently happened at home and in our midst. The Censor—perhaps of necessity—limits the opportuinty of public reference to these. But tales could be told which by a mere plain recital would stir the blood to fury, and to, what is better, iron determination. The records of our hospitals during the present month must be preserved, and in due time made public property, in order that humanity's cry for justice may not remain unsatisfied. A mere passion for revenge is an unworthy sentiment to encourage, but justice stands on a different level and makes a more sustained appeal. No; things have been done which must not be forgotten, and our merchant seamen have shown us the way. In this respect we would harden men's hearts and make them resolute for punishment. Wherever in an open town a non-combatant has been murdered by a German bomb we would set up a memorial so that all men should recall with a sense of bitter contempt the nation which has made the slaying of wounded men and of women and children a part of its systematic policy and ambition. There must be no forgetting, but rather that firm memory which sees things as they really are, and recognises that irrevocably fixed in the foundations of life is the conviction that crime must not escape its penalty.

